# First-line therapy in drug reaction with eosinophilia and systemic symptoms (DReSS): Thinking beyond corticosteroids

**DOI:** 10.3389/fmed.2023.1138464

**Published:** 2023-02-08

**Authors:** Ruud H. J. Verstegen, Elizabeth J. Phillips, David N. Juurlink

**Affiliations:** ^1^Department of Paediatrics, University of Toronto, Toronto, ON, Canada; ^2^Division of Clinical Pharmacology and Toxicology, Department of Paediatrics, The Hospital for Sick Children, Toronto, ON, Canada; ^3^Division of Rheumatology, Department of Paediatrics, The Hospital for Sick Children, Toronto, ON, Canada; ^4^Department of Medicine, Vanderbilt University Medical Center, Nashville, TN, United States; ^5^Department of Dermatology, Vanderbilt University Medical Center, Nashville, TN, United States; ^6^Department of Pathology, Microbiology and Immunology, Vanderbilt University Medical Center, Nashville, TN, United States; ^7^Department of Pharmacology, Vanderbilt University Medical School, Nashville, TN, United States; ^8^Department of Medicine, University of Toronto, Toronto, ON, Canada; ^9^Division of General Internal Medicine, Sunnybrook, Toronto, ON, Canada; ^10^Division of Clinical Pharmacology and Toxicology, Sunnybrook, Toronto, ON, Canada

**Keywords:** DReSS (drug reaction with eosinophilia and systemic symptoms), adverse (side) effects, cyclosporine, targeted therapy, severe cutaneous adverse drug reaction

Drug reaction with eosinophilia and systemic symptoms (DReSS), also known as drug-induced hypersensitivity syndrome (DIHS), carries considerable short- and long-term morbidity, along with a mortality rate of up to 10% (1). Prompt diagnosis, withdrawal of the implicated drug and optimal treatment are crucial to optimize patient outcomes.

Clinicians should maintain a high index of suspicion for DReSS/DIHS in patients who present with a new-onset exanthem and fever within 2 to 6 weeks after starting a new medication (1). This is especially true when clinical features progress or when they are accompanied by facial edema, lymphadenopathy, hematological abnormalities (such as lymphopenia, atypical lymphocytosis or eosinophilia), hepatitis or acute kidney injury, although any organ can be involved. Importantly, eosinophilia is an inconsistent or late finding in DReSS/DIHS, and its absence does not exclude the diagnosis.

The drugs most strongly associated with DReSS/DIHS have not changed over the last 15 years and include antibiotics (particularly vancomycin, trimethoprim-sulfamethoxazole and minocycline), anticonvulsants (principally lamotrigine, carbamazepine and phenytoin), allopurinol, and non-steroidal anti-inflammatory drugs (2). The management of DReSS/DIHS consists of prompt discontinuation of all potential culprit drugs, meticulous supportive care, and immunosuppressive therapy in all but the mildest cases (1).

For decades, systemic corticosteroids have been advocated as first-line agents in patients with moderate to severe DReSS/DIHS, despite a lack of rigorous evidence demonstrating their superiority to other options (3). This recommendation stems at least in part from the familiarity most physicians have in using systemic corticosteroids for other immune-mediated diseases. A variety of dosing strategies are used, ranging from initial intravenous methylprednisolone pulse treatment (10–30 mg/kg/dose) to oral treatment with prednisone (0.5–2 mg/kg/day), tapered over a period of at least 2–3-months, although sometimes considerably longer (1, 3). While the collective medical experience with corticosteroids is extensive, in our view, there are several concerns related to the routine use of corticosteroids in the management of DReSS/DIHS that warrant reassessment of the prevailing “steroids first” treatment paradigm, and consideration of targeted therapies that better address the mechanistic basis of the disease.

DReSS/DIHS appears fundamentally different from other inflammatory conditions (e.g., asthma, arthritis, systemic lupus erythematosus) for which short-term corticosteroids are typically prescribed and effective. For instance, while most inflammatory conditions show rapid improvement upon the initiation of steroids, it is not unusual for symptoms related to DReSS/DIHS to improve slowly or even worsen—regardless of corticosteroid dosing or route of administration. Second, DReSS/DIHS is prone to relapse and is notoriously sensitive to relatively small dose changes. For example, when parenteral steroid therapy is transitioned to oral treatment, or when the daily prednisone dose is tapered by 5 mg, it is not uncommon to observe intensification of symptoms and worsening laboratory markers. More importantly, increased disease activity in DReSS/DIHS may be accompanied by the development of features not initially present at diagnosis, such as involvement of a single new organ (e.g., hepatitis or myocarditis). This is in significant contrast with patients with other inflammatory conditions, who normally tolerate such dosing changes well, and in whom disease flares associated with small dose changes are generally mild. Finally, DReSS/DIHS typically necessitates a protracted course of corticosteroids, often over a period of 2 to 3 months, and often longer, to avoid flares in disease activity. This is significantly longer than schedules used for most other inflammatory conditions. While we recognize that corticosteroids are often able to prevent disease progression and its immediate use may be necessary to manage specific complications of DReSS/DIHS (e.g., hemophagocytic syndrome), corticosteroids are a “blunt instrument” that do not target the pathogenesis of DReSS/DIHS directly.

The disease process of DReSS/DIHS is primarily characterized by the development and activation of drug-specific T cells, along with dysregulation of regulatory T cells (1). In addition, it remains unclear if latent viral reactivation contributes to the clinical phenotype or is a consequence of T-cell activation or immunosuppressive treatment itself. The severity of DReSS/DIHS predicts cytomegalovirus (CMV) reactivation, which subsequently is a marker of DReSS/DIHS severity and mortality (4). Cyclosporine is a calcineurin inhibitor that primarily inhibits the activation and proliferation of T cells by blocking T-cell receptor (TCR)-induced interleukin-2 (IL-2) synthesis and inhibition of TCR signaling. Given this, along with decades of experience in the use of cyclosporine in other T-cell mediated inflammatory diseases, cyclosporine has been increasingly used in the management of recalcitrant DReSS/DIHS (1, 5, 6).

A growing number of case reports describe the use of cyclosporine in DReSS/DIHS. For example, Nguyen et al. described five adults with DReSS/DIHS who were treated with cyclosporine, comparing them to 21 patients who received corticosteroids (5). In this small series, symptom resolution occured quicker in those receiving cyclosporine, resulting in a reduced hospital stay (8.1 vs. 16.2 days) and treatment duration (12.5 vs. 48.5 days). Although it is not possible to draw strong conclusions from these data, in part because there may have been differences in disease severity, their observations align with increasing clinical experience in this area (5, 6).

Setting aside the strong mechanistic rationale and anecdotes of favorable outcomes, a short course of cyclosporine has a far more favorable safety profile compared to a prolonged course of corticosteroids. This is especially the case when using relatively low doses for a short period of time, as is typically implemented for DReSS/DIHS (e.g., 5 mg/kg/day, divided twice daily, orally, for 2–4 weeks). In our experience, patients tolerate short courses of cyclosporine well, especially when compared to those who receive prolonged courses of high-dose corticosteroids.

Given the rarity and sporadic nature of DReSS/DIHS as well as the need for large, coordinated networks to study it, cyclosporine and corticosteroids have not been compared in a controlled clinical trial. However, our experience and that of others (5, 6) have reinforced the observations of more rapid improvement and more favorable tolerability of cyclosporine relative to corticosteroids. While both therapies can be effective, and individual factors such as medical co-morbidities and potential drug-drug-interactions should factor into treatment choices, in our view the balance of potential benefits and harms favors cyclosporine as initial therapy for many patients. For this reason, we believe that cyclosporine should enjoy a more prominent role in the early management of DReSS/DIHS while we await much needed head-to-head comparisons to corticosteroids. In severe cases, corticosteroids can be used in conjunction with it, and then tapered over weeks rather than months.

Besides cyclosporine, there are other targeted therapies that might be considered for the management of DReSS/DIHS. For example, a recent study presented single-cell data from the skin biopsy from an individual with recalcitrant DReSS/DIHS secondary to trimethoprim-sulfamethoxazole. The authors showed upregulation of JAK/STAT markers on T cells, a clinical response to the JAK 1/3 inhibitor tofacitinib, as well as tofacitinib suppression of trimethoprim-sulfamethoxazole-induced CD4+ T-cell proliferation *in vitro* (7). Examples like these give a glimpse of how personalized and mechanistic data might be used to effectively target treatment of DReSS/DIHS.

The management of patients with DReSS/DIHS remains challenging due to a lack of randomized controlled trials to identify the optimal treatment for this uncommon and heterogenous but often severe adverse drug reaction. While the challenges of treating a rare condition such as DReSS/DIHS can and should be addressed to reduce the harm and improve outcomes, the lack of high-quality data need not limit practitioners from considering alternative treatment modalities that have stronger mechanistic rationale and more favorable short-term safety profiles.

## Author contributions

RV wrote the first draft of the manuscript. All authors contributed to the conception of the manuscript and revision and approved the submitted version.
